# Cheese Consumption and Incidence of Dementia in Community-Dwelling Older Japanese Adults: The JAGES 2019–2022 Cohort Study

**DOI:** 10.3390/nu17213363

**Published:** 2025-10-25

**Authors:** Seungwon Jeong, Takao Suzuki, Yusuke Inoue, Eunji Bang, Kentaro Nakamura, Mayuki Sasaki, Katsunori Kondo

**Affiliations:** 1 Faculty of Health Sciences, Niimi University, 1263-2 Nishigata, Niimi 718-8585, Japan; yusuke-inoue@niimi-u.ac.jp; 2 National Center for Geriatrics and Gerontology, 7-430 Morioka, Obu 474-8511, Japan; suzutaka@ncgg.go.jp; 3 Japan Agency for Gerontological Evaluation Study (JAGES Agency), C208, KOIL 16GATE, Block 133-6, 394-11 Shorenji, Kashiwa 277-0814, Japan; e.bang@jages.net; 4 R&D Division, Meiji Co., Ltd., 1-29-1 Nanakuni, Hachioji 192-0919, Japan; kentarou.nakamura@meiji.com (K.N.); mayuki.sasaki@meiji.com (M.S.); 5 Center for Preventive Medical Sciences, Chiba University, 1-33, Yayoicho, Inage-ku, Chiba 263-8522, Japan; team-kk.cpms@chiba-u.jp; 6 Research Department, Institute for Health Economics and Policy, 1-21-19, Toranomon, Minato-ku, Tokyo 101-0001, Japan

**Keywords:** cheese consumption, dementia incidence, community-dwelling older adults, Japan, cohort study, propensity score matching

## Abstract

**Background/Objectives:** Dementia is a growing public health concern in rapidly aging Japan. Dietary factors, including dairy products, have been proposed as modifiable influences on cognitive health, although findings across studies remain inconsistent. This study aimed to examine the association between habitual cheese consumption and incident dementia in a large, population-based cohort of older Japanese adults, and to provide epidemiological evidence regarding its potential preventive role in populations with low baseline dairy intake. **Methods:** We analyzed data from the Japan Gerontological Evaluation Study (JAGES) 2019–2022 cohort, linking survey responses to long-term care insurance (LTCI) certification records. Participants aged ≥65 years without prior LTCI certification were included. Cheese consumption was assessed at baseline and categorized as ≥1 time/week vs. non-consumers. Propensity score matching (PSM) was applied on sociodemographic and health-related covariates. Cox proportional hazards models estimated hazard ratios (HRs) for incident dementia over three years. **Results:** After PSM, 7914 participants were analyzed (3957 consumers; 3957 non-consumers). Baseline covariates were well-balanced. Over 3 years, 134 consumers (3.4%) and 176 non-consumers (4.5%) developed dementia, corresponding to an absolute risk difference of 1.06 percentage points. Cheese consumption was associated with a lower hazard of dementia (HR = 0.76, 95% CI 0.60–0.95, *p* = 0.015). **Conclusions:** Habitual cheese consumption (≥1 time/week) was modestly associated with a reduced 3-year incidence of dementia in older Japanese adults. While the absolute risk reduction was small, these findings are consistent with prior observational evidence linking dairy intake to cognitive health. Further research is warranted to clarify dose–response relationships, cheese subtypes, and underlying mechanisms.

## 1. Introduction

Dementia is a major global public health challenge, with its prevalence expected to increase rapidly in parallel with population aging. The World Health Organization (WHO) has identified dementia as a key public health priority and launched the Global Action Plan on the Public Health Response to Dementia 2017–2025 [1]. According to the WHO’s Global Status Report on the Public Health Response to Dementia, more than 50 million people worldwide were living with dementia in 2021, and this number is projected to more than triple by 2050 [2]. To address this growing burden, WHO has emphasized coordinated global action on prevention, diagnosis, care, and policy development.

Japan, facing one of the world’s fastest aging populations, is experiencing a similar trend. In 2022, dementia affected 12.3% (4.4 million people) of adults aged ≥65 years, and this number is expected to rise to 5.8 million by 2040 [3]. The increasing prevalence of dementia places pressure on medical and long-term care systems and adds psychological and social burdens on families. Because curative treatment remains limited, preventive strategies focusing on modifiable risk factors have become a critical priority.

Among modifiable factors, diet has attracted growing attention in dementia prevention research. Cheese, in particular, contains vitamin K2 [4], bioactive peptides [5], and probiotics [6], which may exert neuroprotective effects through anti-inflammatory and metabolic pathways.

Epidemiological studies have suggested inverse associations between dairy consumption and cognitive decline [7,8]; however, the results remain inconsistent across populations and study designs [9,10].

Given these inconsistencies, we deliberately employed an observational design without active intervention to investigate the relationship between habitual cheese consumption and incident dementia. While this approach has inherent methodological limitations compared with intervention studies, it may better reflect real-world dietary behavior and long-term associations.

This study aimed to examine the prospective association between habitual cheese consumption and incident dementia in a large, population-based cohort of older Japanese adults, and to provide epidemiological evidence regarding its potential preventive role in populations with low baseline dairy intake.

## 2. Materials and Methods

### 2.1. Study Design and Setting

This was a longitudinal, population-based observational study. We used an individual-level cohort dataset created by linking the Japan Gerontological Evaluation Study (JAGES) 2019 “Health and Living” questionnaire with long-term care insurance (LTCI) certification records for fiscal year 2022 provided by participating municipalities. The 2019 survey was an extensive, self-administered questionnaire conducted in 64 municipalities and targeted community-dwelling adults aged ≥65 years who had never been certified for LTCI benefits at baseline.

### 2.2. Participants

**Figure 1:**
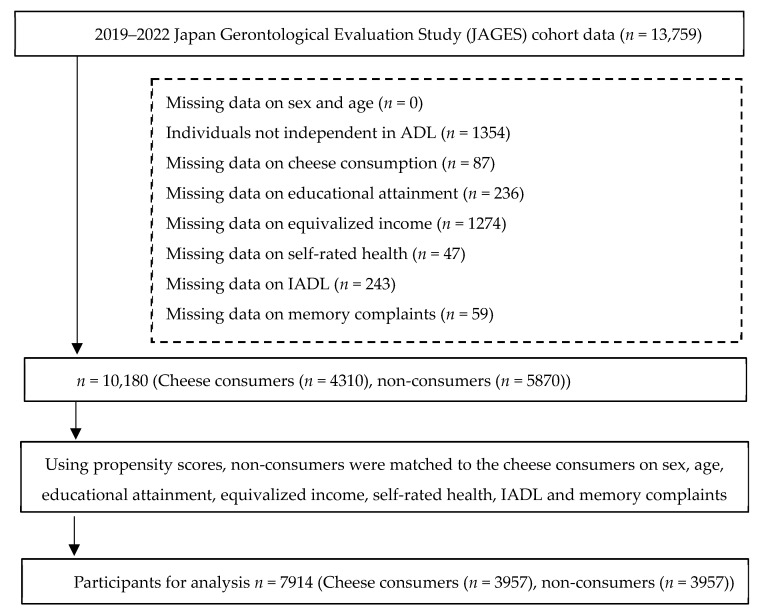
Flow of participant inclusion and exclusion for the analysis of cheese consumption and dementia incidence in the JAGES 2019–2022 cohort.

A flow diagram of participant selection is presented in Figure 1. Of 26,408 valid respondents to the 2019 survey, record linkage with 2022 LTCI certification data and LTCI premium (assessment) files was available in 39 municipalities (*n* = 13,759). We then performed data cleaning according to the following exclusion criteria: (1) missing sex or age, (2) not independent in activities of daily living (ADL), (3) missing data on the exposure (cheese consumption), and (4) missing data on covariates (sex, age, educational attainment, equivalized income, self-rated health, instrumental ADL [IADL], and memory complaints). After exclusions, 10,180 participants remained eligible for propensity score matching (PSM).

To compare those with and without cheese consumption after aligning potential confounders, we conducted 1:1 nearest-neighbor PSM. The final analytic sample after matching was 7914 participants (cheese consumers, *n* = 3957; non-consumers, *n* = 3957).

### 2.3. Variables

#### 2.3.1. Outcome: Dementia Incidence

In Japan, the level of care required for dementia is determined through a standardized process, which includes interviews conducted by municipal officials, written opinions from the patient’s primary care physician, and a national certification algorithm that calculates required care time [11]. Incident dementia was defined based on the first recorded date of dementia in the Long-Term Care Insurance (LTCI) certification system ledger (new entry during follow-up).

#### 2.3.2. Exposure: Cheese Consumption

The primary exposure compared cheese consumers vs. non-consumers. Participants who reported eating cheese ≥ 1 time/week at baseline were classified as cheese consumers, whereas those who reported rarely or never consuming cheese were classified as non-consumers.

#### 2.3.3. Covariates

Covariates included sex, age, educational attainment, equivalized household income, self-rated health, memory complaints, and IADL impairment, defined as having at least one limitation in instrumental activities of daily living (IADL) at baseline.

Equivalized household income was calculated using two variables: (1) the total annual household income during the past year (including pensions), and (2) the number of people living in the household. Participants reported their total household income by selecting one of 15 categories ranging from less than 0.5 million yen to 15 million yen or more. The median value of each income category was then divided by the square root of the household size to obtain the equivalized income.

Educational attainment was assessed by asking: “How many years of formal schooling did you complete?” Response categories included “<6 years,” “6–9 years,” “10–12 years,” “≥13 years,” and “Other.” For this study, educational attainment was categorized as “<6 years,” “6–9 years,” and “≥10 years.”

Self-rated health was assessed by asking: “How would you rate your current health?” Response options were “very good,” “good,” “not very good,” and “poor.” Responses were dichotomized into “good” (very good or good) and “poor” (not very good or poor).

Instrumental activities of daily living (IADL) were assessed using the Tokyo Metropolitan Institute of Gerontology (TMIG) Index of Competence. Participants were asked five questions regarding their ability to: (1) use public transportation, (2) shop for daily necessities, (3) prepare meals, (4) pay bills, and (5) manage deposits and savings. Each item had three response options: “able and do,” “able but do not,” and “unable.” Participants who responded “unable” to one or more items were classified as “IADL impaired,” and those with no limitations were classified as “IADL independent.”

Memory complaints were assessed by asking: “Have you ever been told by others that you often forget things?” with binary response options (“yes” or “no”).

All measures used in this study were based on standardized survey items and indicators officially adopted by the Ministry of Health, Labour and Welfare of Japan.

### 2.4. Bias and Confounding Control

To minimize confounding by socioeconomic and health-related factors, propensity score matching was conducted using logistic regression, including age, sex, education, income, self-rated health, IADL, and memory complaints. Matching used a caliper of 0.2 of the standard deviation of the logit of the propensity score [12]. Additional models adjusted for dietary pattern variables.

### 2.5. Study Size

The study used an available sample from the JAGES cohort; no a priori sample size calculation was performed. However, the analytic sample size was sufficient to detect a modest effect size with adequate precision.

### 2.6. Follow-Up and Censoring

Follow-up began at the 2019 questionnaire date and was scheduled to end at incident dementia or the end of fiscal year 2022. Participants who died or moved out of the municipality during follow-up were censored at the date of death or out-migration. As a sensitivity analysis, we confirmed that results were materially unchanged when these participants were instead treated as right-censored at the date of death or out-migration. Participants with missing data on exposure or covariates were excluded prior to matching.

### 2.7. Statistical Analysis

We first assessed covariate balance after PSM using standardized mean differences (SMDs) and considered SMD < 0.10 to indicate adequate balance [13]. After confirming the balance, baseline characteristics are described by group.

The primary analysis applied a Cox proportional hazards model with incident dementia as the outcome and cheese consumption (consumers vs. non-consumers) as the exposure, reporting hazard ratios (HRs) and 95% confidence intervals (CIs). As a sensitivity analysis, we fitted a multivariable Cox model without PSM. Two-sided *p* < 0.05 was considered statistically significant. Analyses were conducted using Stata SE 16.1 (StataCorp, College Station, TX, USA).

## 3. Results

In this longitudinal analysis, we investigated whether cheese consumption was associated with the incidence of dementia among community-dwelling older adults in Japan over a 3-year follow-up period.

### 3.1. Baseline Characteristics

After propensity score matching (PSM), the final analytic sample included 7914 participants (cheese consumers, *n* = 3957; non-consumers, *n* = 3957). Baseline characteristics were well balanced between the two groups, with all standardized mean differences (SMDs) below the conventional threshold of 0.10, and sex was exactly at 0.10 (the threshold); the remaining covariates ranged from 0.004 to 0.079, indicating successful matching. The proportion of men among consumers was 60.1%, and among non-consumers, it was 56.2%. The age distribution was comparable, with 21.4% versus 26.4% aged 65–69 years and 5.8% versus 6.3% aged 85 years or older, respectively. Educational attainment, equivalized income, self-rated health, instrumental activities of daily living (IADL), and memory complaints were also similarly distributed across groups (Table 1).

### 3.2. Patterns of Cheese Consumption

Among consumers, the majority reported eating cheese 1–2 times per week (72.1%), while smaller proportions reported eating it 3–4 times per week or more than 5 times per week. Regarding cheese type, processed cheese was the most consumed (82.7%), followed by white mold cheese (7.8%) and other varieties (Table 2).

### 3.3. Incidence of Dementia During Follow-Up

During the 3-year follow-up, 134 participants (3.39%) in the consumer group and 176 participants (4.45%) in the non-consumer group developed dementia. This corresponds to an absolute risk difference of 1.06 percentage points, equivalent to approximately 10.6 fewer cases per 1000 participants over a 3-year period among consumers.

**Figure 2:**
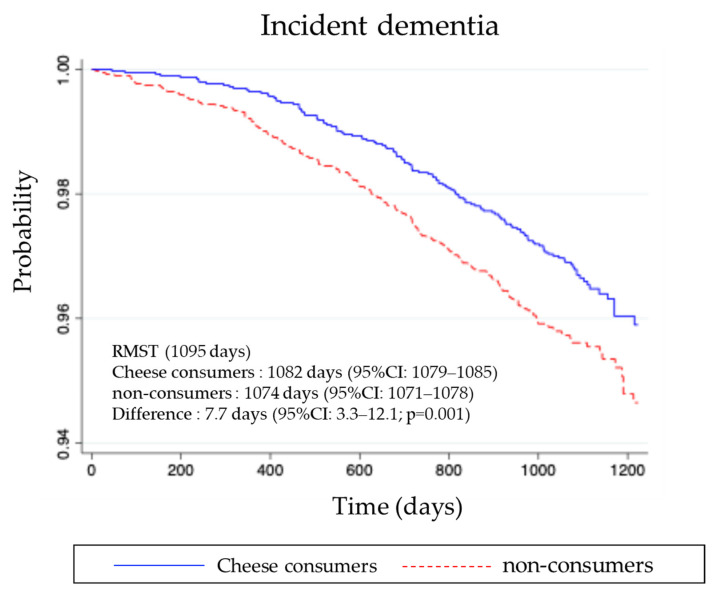
Kaplan–Meier survival curves for dementia incidence among cheese consumers (blue solid line) and non-consumers (red dashed line) after propensity score matching (PSM).

Kaplan–Meier survival curves (Figure 2) demonstrated a significantly lower cumulative incidence of dementia among cheese consumers compared with non-consumers (log-rank test, *p* < 0.05).

Because cheese consumption patterns may reflect broader dietary behaviors, propensity score matching was performed, including socioeconomic factors such as income and education, to minimize confounding. In addition, the frequency of meat or fish, vegetable or fruit intake (≥4 times/week vs. <4 times/week) was included as an indicator of overall dietary pattern in an additional model.

In Model 1, which included only propensity score matching covariates, cheese consumption was associated with a significantly lower risk of dementia (HR = 0.76; 95% CI: 0.60–0.95; *p* = 0.02). In Model 2, we further adjusted for the frequency of meat or fish and vegetable or fruit intake (≥4 times/week vs. <4 times/week). Lower intake frequencies (<4 times/week) of these food groups were more common among non-consumers. Specifically, 19.9% of non-consumers reported vegetable or fruit intake <4 times/week compared with 6.4% of cheese consumers, and 28.3% of non-consumers reported meat or fish intake < 4 times/week compared with 19.9% of cheese consumers. The association remained significant but slightly attenuated (HR = 0.79; 95% CI: 0.63–0.99; *p* = 0.04) (Table 3).

In addition, restricted mean survival time (RMST) analysis revealed that the average dementia-free survival time over 3 years was 1082 days among consumers, compared with 1074 days among non-consumers, representing a difference of approximately 7.7 days.

## 4. Discussion

In this longitudinal cohort study of community-dwelling older Japanese adults, habitual cheese consumption (≥1 time/week at baseline) was associated with a lower risk of incident dementia over a 3-year period. After propensity score matching, the hazard of dementia was 24% lower in consumers than in non-consumers (HR = 0.76; 95% CI: 0.60–0.95). The 3-year cumulative incidence was 3.39% among consumers and 4.45% among non-consumers, corresponding to an absolute risk difference of 1.06 percentage points. These findings are broadly consistent with previous epidemiological evidence suggesting a potential protective association between fermented dairy products and cognitive outcomes [7,8,14,15].

### 4.1. Biological Plausibility

In this cohort study, habitual cheese consumption was associated with a lower risk of incident dementia over a 3-year period. Although the present study did not include biomarker or mechanistic assessments, several nutritional characteristics of cheese may provide a plausible explanation for the observed association. Cheese contains proteins and essential amino acids that support neuronal maintenance, as well as fat-soluble vitamins such as vitamin K2, which plays a role in vascular health and calcium homeostasis [16]. It also provides antioxidants (e.g., selenium, vitamin E) and, when fermented, generates bioactive peptides and probiotic strains that have been implicated in gut–brain axis modulation and inflammation regulation [17,18,19].

In addition, fermented dairy products have been associated with lower risks of cardiovascular and metabolic diseases [20,21], both of which are known risk factors for dementia [22]. While these pathways were not directly assessed in the present study, they represent biologically plausible mechanisms that may partly account for the epidemiological association observed. In our study, most participants consumed processed cheese, which may contain lower levels of bioactive peptides and probiotics compared with fermented cheeses. Therefore, while the biological mechanisms proposed (e.g., vitamin K2, antioxidant effects, gut–brain modulation) are plausible, their relevance to processed cheese may be more limited. This discrepancy highlights the need for future studies examining specific cheese types and their biochemical components in relation to cognitive outcomes.

### 4.2. Epidemiological Evidence Linking Cheese and Cognitive Outcomes

A growing body of epidemiological research has examined the association between cheese consumption and cognitive outcomes, providing a certain level of evidence, although findings vary depending on product type, intake levels, and study design.

One of the most notable studies is the Finnish KIHD cohort, which followed participants for approximately 22 years. This study demonstrated a significant reduction in dementia risk among those with the highest levels of cheese intake, with a 28% lower risk compared to the lowest intake group (HR = 0.72, 95% CI: 0.52–0.99) [23]. This finding is consistent with the results observed in the present study.

In Japan, a cross-sectional study of older women reported that consumption of fermented cheese, such as Camembert, was significantly associated with a lower risk of cognitive decline (OR = 0.448, 95% CI: 0.214–0.936) [7]. The Hisayama study also showed that dairy consumption was associated with a reduced risk of Alzheimer’s disease [24], suggesting that higher dairy intake may be linked to a lower risk of cognitive decline.

On the other hand, heterogeneity in intake levels and outcome assessment across studies has prevented definitive conclusions regarding dose–response relationships, and some studies have reported null or weaker associations [25].

Beyond epidemiological associations, data from the UK Biobank have provided insight into potential neurobiological pathways. Cheese consumption was associated with altered functional connectivity within specific neural networks, and these effects varied according to genetic background, such as APOE4 carrier status [26]. This suggests that the impact of cheese consumption on cognitive health may be modified by individual genetic susceptibility.

Collectively, these findings support a potential protective association between cheese consumption and cognitive outcomes, although variation in intake levels and population characteristics warrants careful interpretation.

### 4.3. Regional Differences and Dose–Response Considerations

Associations between cheese consumption and cognitive outcomes may vary depending on baseline intake levels. Previous studies have suggested that the relationship between dairy intake and cognitive outcomes may vary across regions. In Japan, where baseline cheese intake remains low, smaller increments could correspond to noticeable effects. In contrast, evidence from Western cohorts with substantially higher intake levels has frequently shown weaker or null associations [10], pointing to a potentially non-linear pattern.

In the present study, only consumption frequency—not quantity—was assessed, which limits the ability to evaluate dose–response effects in detail. However, given the overall low cheese intake in Japan, even relatively small differences in consumption frequency may reflect meaningful differences in absolute intake and could partly explain the observed association. Further research is needed to clarify optimal intake levels and potential thresholds for cognitive benefits.

### 4.4. Public Health Implications

The absolute risk reduction observed in this study was modest, equivalent to approximately 10.6 fewer dementia cases per 1000 individuals over 3 years. Although the effect for each person is modest, at a population scale, especially in countries with low cheese intake, such differences could contribute meaningfully to dementia prevention strategies. In Japan, where per capita cheese intake (2.7 kg/person-year, 2019–2021) is considerably lower than in EU countries (>20 kg/person-year) [27]. Incremental increases in intake, when combined with established dementia risk reduction strategies such as vascular risk management and physical activity promotion, could contribute to population-level dementia prevention efforts.

### 4.5. Methodological Strengths and Limitations

Strengths of this study include the use of a large population-based cohort, exclusion of individuals already certified for long-term care at baseline, and propensity score matching to reduce confounding. Dementia onset was identified through administrative long-term care insurance records, facilitating follow-up within the study cohort.”

Several limitations should be noted. First, cheese consumption was assessed only once at baseline, and no repeated dietary measures were available during follow-up. Portion size information was not collected, and although frequency data can serve as a rough proxy for dose, this limits the precision of exposure assessment. Second, major confounding factors such as APOE ε4 genotype were not measured, which may lead to residual confounding. Third, dementia onset was based on administrative records and may not align precisely with clinical diagnosis, and information on dementia subtypes (e.g., Alzheimer’s disease, vascular dementia, dementia with Lewy bodies) was not available. Finally, the study population consisted of older Japanese adults, and generalizability to populations with different dietary habits may be limited.

### 4.6. Future Research

Future studies should clarify optimal intake levels and evaluate dose–response relationships in different populations and regions. Analyses by dementia subtypes are needed to explore potential heterogeneity of effects. Further nutritional trials could clarify how specific nutrients or fermentation-related compounds in cheese contribute to neuroprotective mechanisms. Comparative studies across different intake settings could also enhance the external validity and generalizability of these findings.

## 5. Conclusions

In this population-based cohort of older Japanese adults, habitual cheese consumption (≥1 time/week) was associated with a modestly lower incidence of dementia over three years, even after adjustment through propensity score matching. Although the absolute risk reduction was slight, the findings are consistent with prior evidence suggesting potential cognitive benefits of fermented dairy products. Our findings align with previous evidence suggesting that dairy products may play a supportive role in dementia prevention, though further studies are needed before making definitive recommendations. Future research is warranted to clarify dose–response relationships, cheese subtypes, and underlying mechanisms.

## Figures and Tables

**Table 1:** Baseline characteristics of participants after propensity score matching (PSM).

Variables		Before PSM Cheese Consumers, *n* = 5870 Non-Consumers, *n* = 4310		After PSM Cheese Consumers, *n* = 3957 Non-Consumers, *n* = 3957	
		Cheese Consumers; *n*, (%)	Non-Consumers *n*, (%)	Cheese Consumers, *n*, (%)	Non-Consumers *n*, (%)
Sex	Male	2642 (45.0)	2543 (59.0)	2379 (60.1)	2225 (56.2)
	Female	3228 (55.0)	1767 (41.0)	1578 (39.9)	1732 (43.8)
Age, years	65–69	1470 (25.0)	1122 (26.0)	847 (21.4)	1045 (26.4)
	70–74	1864 (31.8)	1328 (31.0)	1188 (30.0)	1222 (30.9)
	75–79	1492 (25.4)	988 (22.9)	1090 (27.5)	928 (23.5)
	80–84	756 (12.9)	585 (13.6)	603 (15.2)	513 (13.0)
	≥85	288 (4.9)	287 (6.7)	229 (5.8)	249 (6.3)
Equivalized income, million yen	<2.00	2557 (43.7)	2217 (51.4)	1993 (50.4)	1976 (49.9)
	2.00–3.99	2496 (42.5)	1582 (36.7)	1472 (37.2)	1494 (37.8)
	≥4.00	817 (13.9)	511 (11.9)	492 (12.4)	487 (12.3)
Educational attainment, years	<6	12 (0.2)	22 (0.5)	12 (0.3)	13 (0.3)
	6–9	1124 (19.2)	1251 (29.0)	1124 (28.4)	1034 (26.1)
	≥10	4734 (80.7)	3037 (70.5)	2821 (71.3)	2910 (73.5)
Self-rated health	Excellent or Good	5348 (91.0)	3774 (87.6)	3480 (87.9)	3545 (89.6)
	Fair or Poor	522 (8.9)	536 (12.4)	477 (12.1)	412 (10.4)
Instrumental activities of daily living (IADL)	Independent	5548 (94.5)	3847 (89.2)	3635 (91.9)	3626 (91.6)
	Dependent	322 (5.5)	463 (10.7)	322 (8.1)	331 (8.4)
Memory complaints	No	5216 (88.9)	3679 (85.4)	3373 (85.2)	3426 (86.6)
	Yes	654 (11.1)	631 (14.7)	584 (14.8)	531 (13.4)

Baseline characteristics of cheese consumers and non-consumers were compared after propensity score matching (PSM). Covariate balance was assessed using standardized mean differences (SMDs); all <0.1 (sex was exactly at 0.10 (the threshold)), indicating adequate balance.

**Table 2:** Patterns of cheese consumption in the JAGES 2019–2022 cohort.

Variables		Cheese Consumers (*n* = 3957)	
		*n*	%
Frequency of Cheese consumption	>5 times/week	527	13.3
	3–4 times/week	578	14.6
	1–2 times/week	2852	72.1
Types of cheese consumption, ≥1 time/week	processed cheese	3081	82.7
	fresh cheese	144	3.9
	white mold cheese	289	7.8
	blue mold cheese	18	0.5
	other	195	5.2

**Table 3:** Association between cheese consumption and incidence of dementia after propensity score matching (PSM): Cox proportional hazards model.

	Model 1		Model 2	
	HR (95%CI)	*p*	HR (95%CI)	*p*
non-consumers (Ref.)	1		1	
Cheese consumers (*n* = 3957)	0.76 (0.60–0.95)	0.02	0.79 (0.63–0.99)	0.04

Ref.: reference, HR: hazard ratio, CI: confidence interval. In Model 2, adjusted for meat or fish and vegetable or fruit intake frequency (≥4 times/week vs. <4 times/week)

## Data Availability

The data that support the findings of this study are available from the Japan Gerontological Evaluation Study (JAGES) project. Due to ethical and legal restrictions related to participant privacy, the dataset is not publicly available. However, data may be made available upon reasonable request to the JAGES data management committee, subject to review and approval of a research proposal, study protocol, and evidence of ethical approval.
